# Multicenter Evaluation of a PCR-Based Digital Microfluidics and Electrochemical Detection System for the Rapid Identification of 15 Fungal Pathogens Directly from Positive Blood Cultures

**DOI:** 10.1128/JCM.02096-19

**Published:** 2020-04-23

**Authors:** Sean X. Zhang, Karen C. Carroll, Shawna Lewis, Marissa Totten, Peter Mead, Linoj Samuel, Lisa L. Steed, Frederick S. Nolte, Adam Thornberg, Jennifer L. Reid, Natalie N. Whitfield, N. Esther Babady

**Affiliations:** a Division of Medical Microbiology, Department of Pathology, Johns Hopkins University School of Medicine, Baltimore, Maryland, USA; b Microbiology Laboratory, Johns Hopkins Hospital, Baltimore, Maryland, USA; c Department of Medicine, Memorial Sloan Kettering Cancer Center, New York City, New York, USA; d Henry Ford Health System, Detroit, Michigan, USA; e Medical University of South Carolina, Charleston, South Carolina, USA; f GenMark Diagnostics, Inc., Carlsbad, California, USA; g Department of Laboratory Medicine, Memorial Sloan Kettering Cancer Center, New York City, New York, USA; University of Tennessee at Knoxville

**Keywords:** fungemia, candidemia, blood, fungi, *Candida*, GenMark, *Candida auris*, *Cryptococcus*, *Fusarium*, *Rhodotorula*, bloodstream infections

## Abstract

Routine identification of fungal pathogens from positive blood cultures by culture-based methods can be time-consuming, delaying treatment with appropriate antifungal agents. The GenMark Dx ePlex investigational use only blood culture identification fungal pathogen panel (BCID-FP) rapidly detects 15 fungal targets simultaneously in blood culture samples positive for fungi by Gram staining. We aimed to determine the performance of the BCID-FP in a multicenter clinical study. Blood culture samples collected at 10 United States sites and tested with BCID-FP at 4 sites were compared to the standard-of-care microbiological and biochemical techniques, fluorescence in situ hybridization using peptide nucleic acid probes (PNA-FISH) and matrix-assisted laser desorption ionization–time of flight mass spectrometry (MALDI-TOF MS). Discrepant results were analyzed by bi-directional PCR/sequencing of residual blood culture samples. A total of 866 clinical samples, 120 retrospectively and 21 prospectively collected, along with 725 contrived samples were evaluated. Sensitivity and specificity of detection of *Candida* species (C. albicans, C. auris, C. dubliniensis, C. famata, C. glabrata, C. guilliermondii, C. kefyr, C. krusei, C. lusitaniae, C. parapsilosis, and C. tropicalis) ranged from 97.1 to 100% and 99.8 to 100%, respectively. For the other organism targets, sensitivity and specificity were as follows: 100% each for Cryptococcus neoformans and C. gattii, 98.6% and 100% for *Fusarium* spp., and 96.2% and 99.9% for *Rhodotorula* spp., respectively. In 4 of the 141 clinical samples, the BCID-FP panel correctly identified an additional *Candida* species, undetected by standard-of-care methods. The BCID-FP panel offers a faster turnaround time for identification of fungal pathogens in positive blood cultures that may allow for earlier antifungal interventions and includes C. auris, a highly multidrug-resistant fungus.

## Abstract (precis)

Routine identification of fungal pathogens from positive blood cultures by culture-based methods can be time-consuming, delaying treatment with appropriate antifungal agents. The GenMark Dx ePlex investigational use only blood culture identification fungal pathogen panel (BCID-FP) rapidly detects 15 fungal targets simultaneously in blood culture samples positive for fungi by Gram staining. We aimed to determine the performance of the BCID-FP in a multicenter clinical study. Blood culture samples collected at 10 United States sites and tested with BCID-FP at 4 sites were compared to the standard-of-care microbiological and biochemical techniques, fluorescence in situ hybridization using peptide nucleic acid probes (PNA-FISH) and matrix-assisted laser desorption ionization–time of flight mass spectrometry (MALDI-TOF MS).

## INTRODUCTION

Fungemia is a severe form of systemic and invasive fungal infection and delayed diagnosis of fungal bloodstream infections can result in significant increases in mortality. Candidemia, in particular, is one of the leading causes of bloodstream infections in hospital settings, with a crude mortality rate of 40 to 75% (1). Previously, a multicenter study has shown that the mortality rate significantly increased for every hour of delay in diagnosis of candidemia (2). Rapid diagnosis of candidemia is even more crucial in immunocompromised patient populations because of a higher mortality rate in this patient group (3, 4). Conventional culture-based identification methods lack the speed needed to aid in choosing the appropriate antifungal drugs for timely management of patients suffering from these invasive fungal infections.

Three commercially available molecular tools have been applied to rapidly identify *Candida* spp. directly from positive blood culture bottles (without waiting for the growth of the organisms on the subsequent culture media): the *Candida* PNA FISH assay (OpGen) (5, 6), the BioFire FilmArray blood culture identification panel (bioMérieux) (7), and PhenoTest blood culture kit (Accelerate Diagnostics) (8). One major limitation of each method is the lack of broad coverage for fungal pathogen detection, since the former two methods only target five *Candida* species: C. albicans, C. glabrata, C. krusei, C. parapsilosis, and C. tropicalis, and the latter targets only two *Candida* species: C. albicans and C. glabrata.

The ePlex investigational use only (IUO) blood culture identification fungal pathogen (BCID-FP) panel (GenMark Dx) is a fully automated one-step test to detect and identify 15 fungal pathogens directly from positive blood cultures. In this study, we conducted a multicenter evaluation to determine the clinical sensitivity and specificity of the ePlex IUO BCID-FP panel for the rapid detection and identification of fungal pathogens directly from positive blood cultures.

## MATERIALS AND METHODS

### Study design and samples.

Positive blood cultures from patients of all ages and genders were collected at ten hospitals and medical centers from the following nine cities located in the United States: Albuquerque, New Mexico; Baltimore, Maryland; Charleston, South Carolina; Danville, Pennsylvania; Detroit, Michigan (2 sites); Harvey, Illinois; Indianapolis, Indiana; New York City, New York; and San Diego, California.

Two sites prospectively collected samples in 2015 and 2016, and four sites collected samples from July to August 2018. In addition, samples with Gram staining showing fungal organisms were retrospectively collected from nine sites; they were stored in a freezer (≤−20°C) at the collection sites and then shipped in frozen condition to the testing laboratory where they were stored in −70°C conditions before testing. All prospectively and retrospectively collected positive blood culture samples were tested by the standard-of-care testing (comparator method) performed at each site as per standard laboratory procedures. The residual portion of these blood culture samples was deidentified and tested at four clinical sites with the GenMark Dx ePlex IUO BCID-FP Panel. The study was approved by a central Institutional Review Board (IRB) and/or each site’s IRB.

The comparator method(s) included: traditional fungal culture, FDA-cleared matrix assisted laser desorption ionization–time of flight mass spectrometry (MALDI-TOF MS) (i.e., bioMérieux Vitek MS, Bruker Biotyper), microbiological and biochemical tests (i.e., Becton, Dickinson [BD] Phoenix; bioMérieux Vitek 2; Beckman Coulter MicroScan), and PNA-FISH testing. Discordant results between the BCID-FP panel and the comparator method(s) were investigated by running molecular assays to determine the presence or absence of the organism directly in residual blood culture samples. The molecular assays employed PCR amplification targeting genes associated with each fungal target followed by bi-directional sequencing (PCR/sequencing). The molecular assays were validated analytically with precision, limit-of-detection, inclusivity and exclusivity studies using spiked blood culture media and DNA or whole organisms. Descriptions of each gene target, primer sequences, and PCR conditions are provided in Table S1 in the supplemental materials. As part of the comparator method, all prospective samples were tested with PCR/sequencing assays to determine the presence/absence of Candida auris, *Fusarium* (F. dimerum, F. oxysporum, F. sacchari, F. solani, and F. verticillioides), and *Rhodotorula* (R. glutinis and R. mucilaginosa) because not all standard-of-care methods may have initially tested for these organisms on a consistent basis. Due to potential misidentification of C. parapsilosis with other cryptic species within the C. parapsilosis species complex, e.g., C. orthopsilosis and C. metapsilosis, by standard-of-care phenotypic methods (9, 10), samples with Candida parapsilosis identified by standard laboratory procedures were confirmed using the PCR/sequencing assay to determine the comparator method result.

Contrived samples were used to establish additional performance metrics for specific fungal targets due to very low prevalence within the prospectively and retrospectively collected clinical samples. Each target had contrived samples prepared from at least 3 different strains. Contrived samples were prepared by aseptically injecting 3 to 10 ml of human whole blood (BioIVT, Westbury, NY) into a BD BacTec blood culture bottle (Plus Aerobic/F, Myco/F Lytic, or Peds Plus/F). The bottles were then inoculated with conidia or spores (in case of *Fusarium*) from a pure culture of a known organism grown on Sabouraud agar at 30°C between 36 and 72 h. The fungal preparations were generated by diluting conidia or spores in saline to approximately 0.5 McFarlands via optical density at 600 nm (OD_600_) readings, where 0.5 McFarlands is equivalent to approximately 1.0 × 10^6^ CFU/ml for yeast cells at OD_600_ (11, 12). The fungal preparations were used neat or diluted to either 1:10, 1:100, 1:1,000, 1:10,000, 1:20,000, or 1:100,000 and then 100 μl (except for two samples which used either 400 μl or 1 ml) was used to inoculate the bottle containing blood. The inoculum was adjusted based on successful growth and time to detection in preliminary samples. The time to detection varied from 11 h to 5 days for 95% of the contrived samples; the remaining 5% varied from >5 days to 15 days. The contrived sample list is detailed in Table S2.

### GenMark Dx ePlex BCID-FP panel testing.

The BCID-FP panel runs on a single-use cartridge that automates all aspects of nucleic acid testing in combination with electrowetting and GenMark Dx’s eSensor technology based on the principles of competitive DNA hybridization and electrochemical detection (13). The BCID-FP panel identifies the following 15 targeted fungal organisms from positive blood cultures containing fungi: Candida albicans, C. auris, C. dubliniensis, C. famata, C. glabrata, C. guilliermondii, C. kefyr, C. krusei, C. lusitaniae, C. parapsilosis, C. tropicalis, Cryptococcus gattii, C. neoformans, *Fusarium* spp., and *Rhodotorula* spp.

The test consists of a single-use cartridge to be used with the GenMark Dx ePlex instrument and software, in which all steps from sample extraction to detection of target DNA are performed from a positive blood culture. It combines two main technologies: digital microfluidics, or electrowetting, responsible for the movement and transfer of samples and reagents inside the cartridge, and the GenMark Dx eSensor technology for electrochemical detection of target DNA. Nucleic acids are extracted and purified from blood culture samples (magnetic solid-phase extraction) and DNA is then amplified to generate a double-stranded PCR product. Amplification is followed by an exonuclease treatment to generate a single-stranded PCR product, which is mixed with a solution containing complementary signal probes labeled with ferrocene. If target DNA is present, hybridization between the single-stranded PCR product and the signal probes occurs. The solution is then moved to the detection part of the cartridge, the eSensor microarray, consisting of target-specific capture probes attached to gold electrodes. If present, the complex “target DNA/signal probe” hybridizes with the capture probes, leading to the generation of a voltage signal detected by the ePlex instrument. Internal controls monitoring the performance of each step in the process and each amplification reaction are included on each cartridge.

Testing with the BCID-FP panel was done following the manufacturer’s instructions using the materials in the kit. Briefly, after inverting the blood culture bottle several times to mix, 50 μl was aspirated and loaded into the sample port of the BCID-FP panel cartridge and the cap was depressed to close the port. Each cartridge was barcoded and scanned at the ePlex instrument and inserted into an available bay. Upon test completion, the ePlex instrument ejected the cartridge for disposal and a BCID-FP panel report was generated (Fig. S1).

### Statistical methods.

Sensitivity/positive percent agreement (PPA) and specificity/negative percent agreement (NPA) with comparator method results were determined for each targeted fungal organism detected by the BCID-FP panel. Sensitivity/PPA was calculated as 100× number true positive (TP)/(number TP + number false negative [FN]), while specificity/NPA was calculated as 100× number true negative (TN)/(number TN + number false positive [FP]). The two-sided 95% score confidence interval (CI) was calculated for sensitivity/PPA and specificity/NPA.

## RESULTS

### Sample disposition, run/sample accountability, demographic/sample information.

A total of 447 positive blood culture samples were collected prospectively at 6 sites in 2 phases. In phase I, 237 samples were collected at 2 sites and frozen for future testing (prospective frozen samples) from May 2015 through July 2016. In phase II, 210 samples were collected at 4 sites from July through August 2018, were never frozen, and were tested fresh (prospective fresh samples). Of these 447 blood culture samples, 21 (10 from phase I samples, 11 from phase II samples) had a Gram stain result indicating the presence of fungal organisms, representing an overall prevalence of fungemia of 4.7%. Among the 21 cases, 18/21 included organisms targeted by the BCID-FP panel: 29% were caused by C. glabrata, followed by C. albicans (19%), 10% each by C. tropicalis, C. parapsilosis, C. krusei, and 5% each by C. dubliniensis and *Rhodotorula* spp. (Table S3).

A total of 120 positive blood culture samples with Gram stain results showing fungal organisms were retrospectively collected from 9 sites. In addition, 726 samples were contrived with targeted fungal organisms in BD BacTec bottles (Table S2). Taken together, 867 samples were initially tested with the BCID-FP panel, of which 839 yielded valid results for an initial validity rate of 96.8%. After repeat testing of the 28 initially invalid samples, 27 yielded valid results for a final validity rate of 99.9% (866/867). There was one contrived sample with an invalid result after repeat testing, and therefore it was excluded from the evaluation.

For prospective subjects, 67% were male and the mean age for this group was 48.1 years old, where 71% of the prospective patients ranged in age from 18 to 64 years old. Among the retrospective subjects, 57% were male and the mean age for this group was 53.5 years old, where 55% of these patients ranged in age from 18 to 64 years (Table S4).

Ten different blood culture bottle types from three manufacturers (BD [Becton, Dickinson], bioMérieux Inc, and Thermo Fisher Scientific) were used. The majority of the blood culture bottles used in the prospectively collected samples were BacTec PLUS Aerobic/F. For the retrospectively collected samples, predominant usage was of BacTec PLUS Aerobic/F and BacTec Standard/10 Aerobic/F. For the contrived samples, the bottles were mainly BacTec Myco/F Lytic (Table S5).

### BCID-FP panel performance.

Each of the 15 fungal targets on the BCID-FP Panel was tested by a range of 49 to 70 positive samples to determine sensitivity/PPA and a range of 796 to 817 negative samples to determine specificity/NPA (Table 1). For each fungal target, positive or negative samples (comparator results) consisted of prospectively and retrospectively collected clinical blood culture samples as well as contrived samples. Contrived samples were solely used to evaluate the sensitivity for the following fungal targets due to a lack of positive results from the prospective and retrospective sample collections: Candida auris, C. famata, C. guilliermondii, C. kefyr, C. gattii, and *Fusarium* spp.

**TABLE 1:** Clinical performance of ePlex BCID-FP panel with comparator methods^*e*^

Species	Sensitivity/PPA		Specificity/NPA	
	TP/TP + FN	% (95% CI)	TN/TN + FP	% (95% CI)
*Candida albicans*				
Clinical	53/54	98.1 (90.2–99.7)	87/87	100 (95.8–100)
Contrived	13/14	92.9 (68.5–98.7)	710/711	99.9 (99.2–100)
Combined	66/68	97.1 (89.9–99.2)	797/798	99.9 (99.3–100)
*Candida auris*				
Clinical	0/0		141/141	100 (97.3–100)
Contrived	49/49	100 (92.7–100)	676/676	100 (99.4–100)
Combined	49/49	100 (92.7–100)	817/817	100 (99.5–100)
*Candida dubliniensis*				
Clinical	4/4	100 (51.0–100)	137/137	100 (97.3–100)
Contrived	48/48	100 (92.6–100)	677/677	100 (99.4–100)
Combined	52/52	100 (93.1–100)	814/814	100 (99.5–100)
*Candida famata*				
Clinical	0/0		141/141	100 (97.3–100)
Contrived	51/51	100 (93.0–100)	674/674	100 (99.4–100)
Combined	51/51	100 (93.0–100)	815/815	100 (99.5–100)
*Candida glabrata*				
Clinical	43/44 (**45/46**^*a*^)	97.7 (88.2–99.6)	95/97^*a*^ (**95/95**^*a*^)	97.9 (92.8–99.4)
Contrived	16/16	100 (80.6–100)	709/709	100 (99.5–100)
Combined	59/60	98.3 (91.1–99.7)	804/806	99.8 (99.1–99.9)
*Candida guilliermondii*				
Clinical	0/0		141/141	100 (97.3–100)
Contrived	49/50	98.0 (89.5–99.6)	675/675	100 (99.4–100)
Combined	49/50	98.0 (89.5–99.6)	816/816	100 (99.5–100)
*Candida kefyr*				
Clinical	0/0		141/141	100 (97.3–100)
Contrived	51/51	100 (93.0–100)	672/674	99.7 (98.9–99.9)
Combined	51/51	100 (93.0–100)	813/815	99.8 (99.1–99.9)
*Candida krusei*				
Clinical	4/4	100 (51.0–100)	137/137	100 (97.3–100)
Contrived	46/46	100 (92.3–100)	679/679	100 (99.4–100)
Combined	50/50	100 (92.9–100)	816/816	100 (99.5–100)
*Candida lusitaniae*				
Clinical	3/4	75.0 (30.1–95.4)	137/137	100 (97.3–100)
Contrived	45/45	100 (92.1–100)	679/680	99.9 (99.2–100)
Combined	48/49	98.0 (89.3–99.6)	816/817	99.9 (99.3–100)
*Candida parapsilosis*				
Clinical	18/19^*b*^ (**19/20**^*c*^)	94.7 (75.4–99.1)	121/122^*c*^ (**121/121**^*c*^)	99.2 (95.5–99.9)
Contrived	41/41	100 (91.4–100)	684/684	100 (99.4–100)
Combined	59/60	98.3 (91.1–99.7)	805/806	99.9 (99.3–100)
*Candida tropicalis*				
Clinical	5/5 (**6/6**^*d*^)	100 (56.6–100)	135/136 (**135/135**^*d*^)	99.3 (96.0–99.9)
Contrived	45/45	100 (92.1–100)	680/680	100 (99.4–100)
Combined	50/50	100 (92.9–100)	815/816	99.9 (99.3–100)
*Cryptococcus gattii*				
Clinical	0/0		141/141	100 (97.3–100)
Contrived	50/50	100 (92.9–100)	675/675	100 (99.4–100)
Combined	50/50	100 (92.9–100)	816/816	100 (99.5–100)
*Cryptococcus neoformans*				
Clinical	5/5	100 (56.6–100)	136/136	100 (97.3–100)
Contrived	52/52	100 (93.1–100)	673/673	100 (99.4–100)
Combined	57/57	100 (93.7–100)	809/809	100 (99.5–100)
*Fusarium* spp.				
Clinical	0/0		141/141	100 (97.3–100)
Contrived	69/70	98.6 (92.3–99.7)	655/655	100 (99.4–100)
Combined	69/70	98.6 (92.3–99.7)	796/796	100 (99.5–100)
*Rhodotorula* spp.				
Clinical	2/2	100 (34.2–100)	139/139	100 (97.3–100)
Contrived	48/50	96.0 (86.5–98.9)	674/675	99.9 (99.2–100)
Combined	50/52	96.2 (87.0–98.9)	813/814	99.9 (99.3–100)

a C. glabrata was detected by the ePlex BCID-FP panel in two samples that only grew C. lusitaniae (which was also detected by the ePlex BCID-FP Panel). C. glabrata was further detected in the residual of these two samples by PCR/sequencing, thus confirming these two samples were true positive for C. glabrata. These two samples are also listed as Case 6 and 7 in Table 3.

b The false-negative sample is also listed as Case 3 in Table 3.

c C. parapsilosis was detected by the ePlex BCID-FP panel in a sample that only grew C. dubliniensis (which was also detected by the ePlex BCID-FP panel). C. parapsilosis was further detected in the residual of that sample by PCR/sequencing, thus confirming this sample was true positive for C. parapsilosis. This sample is also listed as Case 4 in Table 3.

d C. tropicalis was detected by the ePlex BCID-FP panel in a sample that only grew C. dubliniensis (which was also detected by the ePlex BCID-FP panel). C. tropicalis was further detected in the residual of that sample by PCR/sequencing, thus confirming this sample was true positive for C. tropicalis. This sample is also listed as Case 5 in Table 3.

e PPA, positive percent agreement; NPA, negative percent agreement; TP, true positive; FN, false negative; TN, true negative; FP, false positive; CI, confidence interval.

Overall, test sensitivity/PPA and specificity/NPA were 100% for the following 6 fungal targets on the BCID-FP panel: C. auris, C. dubliniensis, C. famata, C. krusei, C. gattii, and C. neoformans. The sensitivity/PPA for the remaining fungal targets ranged from 96.2% to 100%, and specificity/NPA ranged from 99.8% to 100%. A total of 9 false-negative results were found in the samples containing the following fungal targets: 5 contrived samples each spiked with C. albicans, C. guilliermondii, *Fusarium* spp., and *Rhodotorula* spp. (*n* = 2); 4 retrospectively collected clinical samples each positive for C. albicans, C. glabrata, C. lusitaniae, and C. parapsilosis (Table 2). A total of 9 false-positive results were detected in the following samples: 5 were found to be positive in contrived samples without spiking for C. albicans, C. kefyr (*n* = 2), C. lusitaniae, and *Rhodotorula* spp.; 4 were from retrospectively collected clinical samples that were not identified by comparator methods but were detected by the BCID-FP panel (2 C. glabrata, 1 C. parapsilosis, and 1 C. tropicalis) (Table 2). A discrepancy analysis was performed by running PCR/sequencing for the above fungal targets in these 4 retrospectively collected samples. The target *Candida* spp. were detected by PCR/sequencing, thus the 4 positive results by the BCID-FP panel were deemed to be true positive. After discordant resolution for the 2 C. glabrata, 1 C. parapsilosis, and 1 C. tropicalis, the sensitivity increased to 98.4%, 98.4% and 100%, respectively, for each target.

**TABLE 2:** Summary of discrepant results between the standard-of-care (SOC) testing or spiked organism and the ePlex BCID-FP panel run^*n*^

Species	SOC positive/BCID-FP negative	PCR/sequencing	Interpretation	SOC negative/BCID-FP positive	PCR/sequencing	Interpretation
*C. albicans*						
Clinical sample (Retrospective)	1	Positive for *C. albicans*	False negative			
Contrived sample	1^*a*^	ND	False negative	1^*b*^	ND	False positive
*C. glabrata*						
Clinical sample (Retrospective)	1^*c*^	Negative for *C. glabrata*	Indeterminate	2^*d*^	Positive for *C. glabrata*	True positive
*C. guillermondii*						
Contrived sample	1^*e*^	ND	False negative			
*C. kefyr*						
Contrived sample				2^*f*^	ND	False positive
*C. lusitaniae*						
Clinical sample (Retrospective)	1	Positive for *C. lusitaniae*	False negative			
Contrived sample				1^*g*^	ND	False positive
						
*C. parapsilosis*						
Clinical sample (Retrospective)	1^*h*^	Positive for *C. parapsilosis*	False negative	1^*i*^	Positive for *C. parapsilosis*	True positive
*C. tropicalis*						
Clinical sample (Retrospective)				1^*j*^	Positive for *C. tropicalis*	True positive
*Fusarium* spp.						
Contrived sample	1^*k*^	ND	False negative			
*Rhodotorula* spp.						
Contrived sample	2^*l*^	ND	False negative	1^*m*^	ND	False positive
**Total**	**9**			**9**		

a The sample was spiked with C. albicans ATCC10231. It was flagged positive on day 6 but was negative by the BCID-FP panel.

b The sample was spiked with C. dubliniensis ATCCMYA-578. C. dubliniensis was correctly detected by the BCID-FP panel, but the sample was also positive for C. albicans and C. kefyr (same sample discussed in footnote *f*).

c The sample grew C. albicans, C. glabrata, and C. dubliniensis. The BCID-FP panel detected C. albicans, C. dubliniensis, but not C. glabrata. Subsequently, C. glabrata was not detected in the residual of that sample by PCR/sequencing. This sample is also listed as Case 1 in Table 3.

d These two samples are also listed as Case 6 and 7 in Table 3.

e The sample was spiked with C. guilliermondii ATCC90198. It was flagged positive on day 2 but was negative by the BCID-FP panel.

f One sample was spiked with C. dubliniensis ATCCMYA-578. C. dubliniensis was correctly detected by the BCID-FP panel, but the sample was also positive for C. kefyr and C. albicans (same sample discussed in footnote *b*). The other sample was spiked with C. auris CDC number 0390. C. auris was correctly detected by the BCID-FP panel, but the sample was also positive for C. kefyr.

g The sample was spiked with C. neoformans ATCC14116. C. neoformans was correctly detected by the BCID-FP panel, but the sample was also positive for C. lusitaniae.

h This sample is also listed as Case 3 in Table 3.

i This sample is also listed as Case 4 in Table 3.

j This sample is also listed as Case 5 in Table 3.

k The sample was spiked with Fusarium dimerum CBS110317. It was flagged positive on day 3 but was negative by BCID-FP Panel.

l Two contrived samples were each spiked with Rhodotorula mucilaginosa ATCC66034 and *R. mucilaginosa* ATCC9449 and flagged positive on day 3 and day 6, respectively, but were negative by BCID-FP Panel.

m The sample was spiked with C. auris CDCnumber 0389. C. auris was correctly detected by BCID-FP Panel, but the sample was also positive for *Rhodotorula*.

n ND, not done.

A total of 8 cases of mixed fungal infections were detected either by comparator methods or by the BCID-FP panel among the 141 prospectively and retrospectively collected clinical samples (Table 2). Case numbers 1, 2, 3, and 8 were mixed infections detected by the comparator methods. Case number 8 contained only fungal pathogens that are not included on the BCID-FP panel (i.e., C. metapsilosis and Trichosporon asahii) which resulted in no targets being detected on the BCID-FP panel, as expected. Case number 1 was a coinfection mixed with C. albicans, C. glabrata, and C. dubliniensis. The BCID-FP panel was able to detect C. albicans and C. dubliniensis, but not C. glabrata. Subsequent PCR/sequencing was not able to confirm the presence of C. glabrata in that sample, rendering an inconclusive evaluation result. In case number 2, the BCID-FP panel was able to detect both C. albicans and C. parapsilosis. In case number 3, the BCID-FP panel was able to detect C. albicans but failed to detect C. parapsilosis. Case numbers 4 to 7 were positive for a single fungal target by the comparator methods. The BCID-FP panel was able to detect not only the single target but also an additional fungal target in each of these 4 cases, as described in the previous paragraph. These additional fungal targets were confirmed by PCR/sequencing results, indicating true coinfections detected by the BCID-FP panel (Table 2).

## DISCUSSION

One of the highest risk factors for mortality for patients with candidemia is time to diagnosis; therefore, rapid, accurate diagnosis is critical to improving patient care outcome (2, 14). The ePlex BCID fungal pathogen panel is currently the only rapid, commercial panel that detects a large number of fungal pathogens (up to 15 pathogens) directly in patients with positive blood cultures. The BCID-FP panel has a straightforward easy-to-use workflow with hands-on time of less than 2 min to load each sample into the cartridge and a run time of approximately 100 min on the ePlex system, a scalable (3 to 24 bays) random-access instrument.

Our multicenter study showed that the ePlex BCID-FP panel exhibited 100% sensitivity and specificity for 6 fungal targets (C. auris, C. dubliniensis, C. famata, C. krusei, C. gattii, and C. neoformans) and a range of sensitivity of 96.2% to 100% and specificity of 99.8% to 100% for the remaining fungal targets before resolution of discordant results. While the ePlex BCID-FP panel missed the detection of fungal targets in 5 contrived samples and 4 retrospective clinical samples (Table 2), the panel did detect additional fungal targets in 4 cases that were missed by the standard-of-care testing, in turn delivering a faster set of complete results to the clinicians responsible for patient management so that appropriate treatment can be initiated without delay. For example, the standard-of-care tests only detected C. lusitaniae in case numbers 6 and 7 of mixed fungal infections (Table 3). The ePlex BCID-FP panel detected additional C. glabrata in both cases, which could have allowed the more appropriate choice of echinocandin over fluconazole as per current clinical practice guidelines for the management of candidiasis (15).

**TABLE 3:** Detection of mixed fungal organisms by the ePlex BCID-FP panel in positive blood cultures (prospective/retrospective clinical samples)

Case no.	SOC testing^*a*^	BCID-FP	PCR/sequencing	Interpretation
1	*C. albicans*	*C. albicans*		
	*C. glabrata*		Negative	Inconclusive
	*C. dubliniensis*	C. dubliniensis		
2	*C. albicans*	*C. albicans*		
	*C. parapsilosis*	*C. parapsilosis*		
3	*C. albicans*	*C. albicans*		
	*C. parapsilosis*		*C. parapsilosis*	BCID-FP false negative
4	*C. dubliniensis*	*C. dubliniensis*		
		*C. parapsilosis*	*C. parapsilosis*	BCID-FP true positive
5	*C. dubliniensis*	*C. dubliniensis*		
		*C. tropicalis*	*C. tropicalis*	BCID-FP true positive
6	*C. lusitaniae*	*C. lusitaniae*		
		*C. glabrata*	*C. glabrata*	BCID-FP true positive
7	*C. lusitaniae*	*C. lusitaniae*		
		*C. glabrata*	*C. glabrata*	BCID-FP true positive
8	*C. metapsilosis*	Off-panel^*b*^		
	*Trichosporon asahii*	Off-panel		

a SOC, standard-of-care.

b Off-panel, the indicated fungal target is not listed in the ePlex BCID-FP panel.

Importantly, BCID-FP is the only FDA-cleared rapid molecular panel that contains C. auris, which is an emerging multidrug-resistant fungal pathogen that has been reported to cause high mortality and nosocomial outbreaks in hospital settings (16–18) and has recently been added to the CDC’s Antimicrobial Resistance Urgent Threat list. Over 60% of patients infected by C. auris developed bloodstream infection with a mortality rate reaching up to 60% (19). Rapid detection of C. auris in blood cultures cannot only result in early initiation of an appropriate antifungal regimen, (i.e., echinocandins due to the pathogen’s high resistance rate to azoles) (19, 20), but can also help prevent further spread of this nosocomial multidrug-resistant organism in health care facilities. A large, multi-institution outbreak of C. auris highlighted the clinical importance of its rapid identification, as transmission occurs primarily among patients with extensive health care exposure and, much like Clostridioides difficile, C. auris remains viable on inanimate objects for 7 to 14 days, longer in a nonculturable state, contributing to its nosocomial transmission (21–23). Although a positive C. auris result has clear epidemiological impact, a negative result for C. auris is also highly valuable for assisting hospital infection control to rule out this nosocomial pathogen, due to the BCID-FP panel’s high specificity for this organism.

The ePlex BCID-FP panel contains 2 non-*Candida* yeasts, *Cryptococcus* and *Rhodotorula*. Although bloodstream infections caused by these yeasts are less common than for *Candida* spp. (24, 25), annually less than 10,000 cases compared to 25,000 cases in the United States, rapid and accurate detection of these fungi are paramount because they contribute to a higher mortality rate and antifungal regimens are very different from candidemia (26, 27). For example, echinocandins are the most active category of antifungal agents against *Candida* spp., but they have no activity against *Cryptococcus* and *Rhodotorula* (27, 28).

Moreover, the ePlex BCID-FP panel is the only commercial panel that also targets *Fusarium* spp., the most common filamentous fungus frequently isolated from patients’ blood cultures (29). The broad coverage of the *Fusarium* target covers the most common and medically important *Fusarium* spp., including F. solani, F. oxysporum, F. verticillioides, F. dimerum, and F. sacchari. Disseminated fusariosis occurs most commonly in immunocompromised patients, particularly those with hematological malignancies, and stem cell transplant patients with prolonged and profound neutropenia and/or severe T-cell immunodeficiency (29). About 60 to 70% of these patients developed a *Fusarium* bloodstream infection and in this patient population the intrinsic resistance of *Fusarium* spp. to most antifungal agents results in high mortality rates (30). Rapid identification of *Fusarium* in these patients would aid in the initiation of proper antifungal treatment that is different from treatment of yeast infection, especially in persistently neutropenic patients with disseminated disease, where the mortality rate approaches 100% (29, 31).

In summary, the ePlex BCID-FP panel, which has recently been cleared by the FDA, contains the largest breadth of fungal targets and proved to be an accurate, easy-to-use multiplex molecular tool suitable for clinical laboratories to detect common fungal pathogens causing bloodstream infections more rapidly than traditional and conventional microbiological methods.

## Supplementary Material

Supplemental file 1

Supplemental file 2
