# Supplementary material for: Multicenter Evaluation of a PCR-Based Digital Microfluidics and Electrochemical Detection System for the Rapid Identification of 15 Fungal Pathogens Directly from Positive Blood Cultures
Source: J Clin Microbiol. 2020 Apr 23;58(5):e02096-19. doi: 10.1128/JCM.02096-19 (PMC7180249; doi:10.1128/JCM.02096-19)
Supplement: Supplemental file 1 [file JCM.02096-19-s0001.pdf]

Supplementary Table 1. Description of each gene target used in the PCR/sequencing identification assay*

Pathogen Target	Molecular Target	Description	Primer Sequence (5′–3′)	Size
Candida albicans	RPB1	DNA-directed RNA polymerase II subunit rpb1	F: M13–TCAGTGGCAAAAATAGAGTATCCTG<br>R: M13–CATCTCTACGAATAGTTGGTTGTGG	430
Candida auris	GABA	gamma-aminobutyric acid (GABA) A receptor	F: GGATACCGAGGAGATCATCCAG<br>R: CATTTTCCTTGACGTGGTCAGG	377
Candida dubliniensis	HWP1	Hyphal wall protein 1	F: M13–CCACTCCAGAAGCTTCCACT<br>R: M13–CCAAAAAAAGAACAGACACGGAT	485
Candida famata	GAPDH	Glyceraldehyde-3-phosphate dehydrogenase	F: M13–ATGATGACATCATTAGACATTCGGATT<br>R: M13–TTTCCGGCAACTTGACTTCTG	384
Candida glabrata	RPB1	DNA-directed RNA polymerase II subunit rpb1	F: M13–CAAGCAATTCAAATCAAGGATCCA<br>R: M13–CCTTCAAGATATCTGCCAATTTGAAT	445
Candida guilliermondii	FKS1	Beta-1,3-glucan synthase catalytic subunit 1	F: M13–GAATCAAGCACTGTGAATACTACCAG<br>R: M13–CAACGAGATAAGATGGCGACAG	488
Candida kefyr	EIF-2α	Eukaryotic Translation Initiation Factor 2 Subunit Alpha	F: M13–GCAGTTATGGATGCTGCTCTATTA<br>R: M13–TCTTACCTTGATCGTCCTTAGTAACT	477
Candida krusei	CS1	Citrate synthase 1	F: M13–TTGCTGAATTGTTACCTGGCTG<br>R: M13–CCTTCGCGAATTTGGATTCTGA	441
Candida lusitaniae	FKS1	Beta-1,3-glucan synthase catalytic subunit 1	F: M13–CTGTGTTTTCTCCATTGGACGT<br>R: M13–GAAAATTGATCGTCTTCCCTGATGATC	504
Candida parapsilosis	RPB1	DNA-directed RNA polymerase II subunit rpb1	F: M13–GGGTGATGATGTCATTAACTACAAG<br>R: M13–CAACTTCTTTGCTTGGTCACGAG	418
Candida orthopsilosis	RPB1	DNA-directed RNA polymerase II subunit rpb1	F: M13–TGGTGATGATGTGAMTAGTTAYAAAG<br>R: M13–CAAYTTCTTAGCTTGGTCACGAG	418
Candida metapsilosis	RPB1	DNA-directed RNA polymerase II subunit rpb1	F: M13–AGGTGAAGATRTYRCTGGATACAAG<br>R: M13–CAACTTCTTRGCTTGGTCACGMG	418
Candida tropicalis	SADH	Alcohol dehydrogenase	F: M13–GGTTTATTGCCAAGATCAAGAAATGT<br>R: M13–TTTCCAGTGGAGTCAATAAACGAC	333
Cryptococcus	RNA Pol	RNA polymerase	F: M13–CCTCTTGGYGAGAACTGGAGAC	387

			R: M13–GCCTTGGCAATCTTCCTCAACTC	
Fusarium	CAP10	Capsular associated protein	F: M13–GATCTACCAGTGCGGTGGTAT	410
			R: M13–GAGGAGTCTCGAACTTCCAGAG	
Rhodotorula	MF1	Anthocyanin methyltransferase	F: M13–TCTGCYATCCCATGGATTGG	314
			R: M13–TTGTCACTGTGTCCYAGGTAG	

*Sanger sequencing assays were developed to detect and differentiate fungi present in bloodstream infections. The PCR primers were designed to detect clinically relevant strains, and they incorporate M13 universal sequencing tags. All PCR reactions utilized a primer annealing temperature of 58 °C and consisted of 40 cycles. With the exception of *Fusarium*, all assays used the Qiagen HotStarTaq Master Mix. Amplification of Fusarium was complicated by the presence of homopolymer tracts that cause polymerase slippage, resulting in poor quality sequencing results; therefore, this assay utilized the New England Biosciences Phusion High-Fidelity Polymerase, a proof-reading polymerase fused to a nonspecific dsDNA binding domain that enhances read-through of the mononucleotide repeat regions. Following PCR, amplicons were visualized by gel electrophoresis to verify successful amplification and then purified using Exonuclease I (Exo I) and Shrimp Alkaline Phosphatase (SAP). Each amplicon was sequenced with M13 primers using the BigDye Terminator v3.1 Cycle Sequencing Kit to yield 2x bi-directional coverage of at least 200 bp. Sequence reads were assembled using Sequencher software to generate a bi-directional consensus sequences. The resulting consensus sequences were then identified using the NCBI Basic Local Alignment Search Tool (BLAST).

Supplementary Table 2. Contrived sample list *

Fungal Organism	Strain	Number Tested	Dilution (1 to x) (min-max)	Spiked-in CFUs/mL (min-max) [a]	Time to Flag Positive [b] (DD:HH:MM) (min-max)
Candida albicans	ATCC10231	2	10 - 100	24.83 - 279.38	00:18:12 - 05:18:26 [c]
Candida albicans	ATCC14053	2	100	24.83 - 27.05	01:05:08 - 02:02:21
Candida albicans	ATCC24433	2	10 - 10000	0.26 - 239.47	00:19:12 - 02:23:09
Candida albicans	ATCC90028	5	1 - 100	19.45 - 2350.33	00:16:07 - 04:10:44
Candida albicans	ATCCMYA-4441	3	10 - 10000	0.25 - 243.90	00:19:12 - 01:15:08
Candida auris	ATCC10913	1	10	235.03	00:20:09
Candida auris	ATCC12372	1	10	230.60	00:18:19
Candida auris	ATCC12766	1	10	248.34	00:17:09
Candida auris	CBS10913	3	10	199.56 - 234.41	00:17:08 - 00:22:12
Candida auris	CBS12372	3	10	234.41 - 284.29	00:16:08 - 00:21:03
Candida auris	CBS12373	2	10	186.25 - 284.29	00:21:08 - 01:00:05
Candida auris	CBS12766	3	10	199.50 - 244.39	00:14:08 - 00:17:43
Candida auris	CBS12767	3	10	208.43 - 248.34	00:16:06 - 00:16:09
Candida auris	CBS12768	2	10	186.25 - 212.86	00:16:08 - 00:17:09
Candida auris	CDC#0385	5	10 - 100	26.61 - 309.23	00:12:30 - 01:12:11
Candida auris	CDC#0386	5	10 - 100	18.18 - 314.86	00:12:30 - 01:11:11
Candida auris	CDC#0387	5	10 - 100	26.61 - 243.90	00:12:30 - 00:22:10
Candida auris	CDC#0388	5	10 - 100	24.83 - 279.30	00:12:30 - 01:00:10
Candida auris	CDC#0389	5	10 - 100	23.06 - 294.26	00:12:30 - 00:22:10
Candida auris	CDC#0390	5	10 - 100	22.62 - 301.55	00:13:00 - 01:02:10
Candida dubliniensis	ATCCMYA-577	6	10 - 100	28.93 - 283.81	00:17:10 - 00:20:07
Candida dubliniensis	ATCCMYA-578	12	10	186.25 - 2434.78	00:14:03 - 00:20:14
	ATCCMYA-579	12	10 - 100000	0.02 - 257.21	00:17:30 - 04:09:43
	ATCCMYA-582	13	10 - 20000	0.11 - 257.21	00:17:04 - 04:08:03
	NCPF3949	5	10 - 100	23.06 - 243.90	00:18:03 - 01:14:06

Candida famata species complex					
Debaryomyces fabryi	CBS789	21	10	195.12 - 310.42	01:22:15 - 14:18:40 [d]
Candida famata	CBS1961	3	10	243.90 - 274.31	01:04:14 - 01:11:07
Debaryomyces subglobosus	CBS1796	27	10 - 100000	0.01 - 301.55	01:01:03 - 04:18:32
Candida glabrata	128-4058	1	100	21.73	01:16:04
Candida glabrata	128-4072	1	100	31.42	03:17:40
Candida glabrata	ATCC15126	2	100	20.84 - 21.29	00:22:08 - 00:23:02
Candida glabrata	ATCC15545	2	100 - 10000	0.23 - 19.96	01:01:11 - 01:18:09
Candida glabrata	ATCC2001	1	100	22.17	01:09:04
Candida glabrata	ATCC66032	4	1 - 100000	0.02 - 2838.14	00:11:05 - 03:13:32
Candida glabrata	ATCC90030	2	1 - 100	19.07 - 2616.41	00:11:06 - 00:23:11
Candida glabrata	ATCCMYA-2950	3	1 - 100	15.52 - 3148.56	01:02:11 - 02:18:00
Candida guilliermondii	ATCC22017	13	10 - 10000	0.23 - 301.55	00:16:58 - 03:21:50
Candida guilliermondii	ATCC6260	12	10 - 10000	0.25 - 279.38	00:17:58 - 01:05:07
Candida guilliermondii	ATCC90197	10	10 - 100	23.95 - 288.25	00:16:58 - 00:23:10
Candida guilliermondii	ATCC90198	9	10 - 100	22.62 - 305.99	00:19:58 - 01:07:20
Candida guilliermondii	ATCC90199	6	10 - 100	23.95 - 270.51	00:18:07 - 01:00:05
Candida kefyr	ATCC204093	4	100 - 10000	0.29 - 30.16	00:15:03 - 01:12:06
Candida kefyr	ATCC2512	10	10 - 100	17.74 - 283.81	00:15:04 - 01:10:09
Candida kefyr	ATCC4135	13	10 - 100	18.18 - 266.08	00:14:04 - 00:23:09
Candida kefyr	ATCC66028	12	10 - 100	19.96 - 274.94	00:13:04 - 00:22:18
Candida kefyr	ATCC8553	12	1 - 100	19.51 - 2971.18	00:14:06 - 05:22:11 [e]
Candida krusei	ATCC14243	8	10 - 100	17.29 - 261.64	00:16:04 - 01:03:08
	ATCC22985	9	10 - 10000	0.22 - 270.51	00:14:04 - 03:01:16
Candida krusei	ATCC28870	9	10 - 10000	0.43 - 279.38	00:13:04 - 02:00:49
Candida krusei	ATCC32196	10	10 - 10000	0.35 - 270.51	00:14:03 - 02:14:30
Candida krusei	ATCC34135	10	10 - 10000	0.35 - 301.55	00:13:09 - 03:02:30
Candida lusitaniae	ATCC26287	6 [f]	10 - 100	25.28 - 310.42	00:14:10 - 01:01:08
Candida lusitaniae	ATCC34449	10	10 - 100	24.83 - 310.42	00:14:00 - 01:03:19
Candida lusitaniae	ATCC42720	9	10 - 100	18.63 - 288.25	00:14:00 - 01:00:03
Candida lusitaniae	ATCC66035	11	10 - 100	23.06 - 328.16	00:14:07 - 01:23:08

Candida lusitaniae	ATCCMYA-766	10	10 - 100	21.29 - 288.25	00:16:00 - 01:19:08
Candida parapsilosis	ATCC22019	11	10 - 100	19.96 - 266.08	00:19:09 - 03:01:16
Candida parapsilosis	ATCC28474	5	10 - 100	18.18 - 208.82	02:13:30 - 03:07:17
Candida parapsilosis	ATCC28475	10	10 - 100	19.07 - 230.60	00:18:19 - 02:18:17
Candida parapsilosis	ATCC58895	7	10 - 100	26.16 - 243.90	00:17:08 - 02:06:11
Candida parapsilosis	ATCC90018	8	10 - 100	18.63 - 270.51	00:19:09 - 01:06:15
Candida tropicalis	ATCC1369	9	10 - 10000	0.25 - 270.51	00:12:39 - 02:02:10
Candida tropicalis	ATCC13803	12	10 - 10000	0.24 - 248.34	00:13:07 - 01:11:05
Candida tropicalis	ATCC201380	9	10 - 10000	0.24 - 270.51	00:14:06 - 02:01:10
Candida tropicalis	ATCC201381	7	10 - 10000	0.35 - 203.99	00:15:08 - 01:22:05
Candida tropicalis	ATCC750	8	10 - 10000	0.25 - 257.21	00:15:07 - 02:00:07
Cryptococcus gattii	ATCC14248	11	10 - 100	17.74 - 239.47	01:06:00 - 01:23:09
	ATCC76108	12	10 - 100	17.74 - 266.08	01:04:07 - 02:19:11
	ATCCMYA-4138	10	10 - 100	17.74 - 297.12	01:00:07 - 07:23:21 [g]
	ATCCMYA-4560	8	10 - 100	18.63 - 270.51	01:05:00 - 09:02:24 [g]
Cryptococcus gattii	ATCCMYA-4877	9	10 - 100	17.29 - 288.25	01:05:07 - 06:00:18 [g]
Cryptococcus neoformans var. *grubii*	ATCC14116	9	1 - 100	17.29 - 2217.29	01:09:06 - 01:22:12
Cryptococcus neoformans var. grubii	ATCC208821(H99)	8	10 - 100	18.63 - 292.68	01:07:00 - 03:00:17
Cryptococcus neoformans var. grubii	ATCC90112	7	10 - 100	19.51 - 257.21	01:07:00 - 02:02:12
Cryptococcus neoformans var. grubii	NCPF8195	7	10 - 100	19.51 - 235.03	01:13:03 - 02:01:11
Cryptococcus neoformans var. grubii	NCPF8299	3	10 - 100	25.72 - 279.38	01:06:07 - 01:17:12
Cryptococcus neoformans var. grubii	NCPF8357	3	10 - 100	23.95 - 270.51	01:06:08 - 01:19:12
Cryptococcus neoformans var. *neoformans*	ATCC34873	3	10	190.69 - 261.64	01:14:17 - 01:20:12
Cryptococcus neoformans var. neoformans	ATCC36556	3	10	230.60 - 301.55	02:08:09 - 02:12:17
Cryptococcus neoformans var. neoformans	ATCCMYA-565	9	10 - 100	17.29 - 283.81	01:06:00 - 02:12:14
Fusarium dimerum	CBS108944	3	100000	2.26	01:13:09 - 01:19:05
Fusarium dimerum	CBS110317	9	10000 - 100000	2.35 - 3.44	01:21:09 - 08:12:17 [h]
Fusarium dimerum	CBS116520	3	100000	3.86	01:11:07 - 01:17:05
Fusarium oxysporum	CBS116611	1	1000	82.93	11:18:11

Fusarium sacchari	ATCC24379	10	10 - 10000	1.86 - 1862.53	02:04:17 - 04:18:15
	CBS119828	11	10 - 10000	13.46 - 13458.98	01:07:59 - 03:15:00
Fusarium solani	ATCC36031	11	10 - 10000	3.44 - 3436.81	01:22:11 - 03:03:30
Fusarium verticillioides	CBS100312	11	10 - 10000	6.27 - 6274.94	01:19:08 - 02:11:00
	ATCC38159	11	10 - 10000	4.57 - 4567.63	02:14:12 - 03:14:00
Rhodotorula glutinis [i]	ATCC32765	3	NA	N/A	NA
Rhodotorula glutinis	ATCC32766	3	NA	N/A	NA
Rhodotorula glutinis	ATCC96365	4	NA	N/A	NA
Rhodotorula mucilaginosa	ATCC66034	21	1 - 1000	2.04 - 2439.02	01:08:09 - 02:18:06
Rhodotorula mucilaginosa	ATCC9449	19	10	190.69 - 274.94	01:01:05 - 11:11:30 [j]

* A total of 678 contrived samples are listed; 47 negative contrived samples that are not included in this supplementary table as they did not contain organisms targeted by the BCID-FP Panel.

[a] Volume spiked was 100uL except 1 replicate of *C. dubliniensis* ATCCMYA-578 (1 mL) and 1 replicate of *F. verticillioides* ATCC38159 (400 uL).

[b] The majority of contrived samples were inoculated into the BacTec Myco/F lytic bottles (640). There were 39 samples inoculated in BacTec Plus Aerobic/F bottles, and 46 in BacTec Peds Pus/F bottles.

[c] One replicate of the strain with 1:100 dilution took greater than 5 days to ring positive

[d] Eleven replicates of the strain took greater than 5 days to ring positive.

[e] One replicate of the strain took greater than 5 days to ring positive.

[f] One of these replicates was a final, invalid result after ePlex BCID-FP testing, so this result was not included in the performance table (Table 2). There were 46 *C. lusitaniae* contrived samples tested but only 45 had valid results to be included.

[g] One replicate of the strain took greater than 5 days to ring positive

[h] Six replicates of the strain took greater than 5 days to ring positive.

[i] Contrived using quantified glycerol stocks (not grown in blood culture system).

[j] Twelve replicates of the strain took greater than 5 days to ring positive.

Supplementary Table 3. Expected Value (Positivity Rate [%]) for the BCID-FP Panel by Age Group
(Prospective Samples)

Target	All Ages (N=21)*	Age <1 (N=1)	Age 1-17 (N=2)	Age 18-44 (N=4*)	Age 45-64 (N=11*)	Age 65-84 (N=2)	Age 85+ (N=1*)
Candida albicans	4 (19.0)	1 (100)	0	0	2 (18.2)	1 (50.0)	0
Candida auris	0	0	0	0	0	0	0
Candida dubliniensis	1 (4.8)	0	0	0	1 (9.1)	0	0
Candida famata	0	0	0	0	0	0	0
Candida glabrata	6 (28.6)	0	1 (50.0)	1 (25.0)	3 (27.3)	1 (50.0)	0
Candida guilliermondii	0	0	0	0	0	0	0
Candida kefyr	0	0	0	0	0	0	0
Candida krusei	2 (9.5)	0	0	0	2 (18.2)	0	0
Candida lusitaniae	0	0	0	0	0	0	0
Candida parapsilosis	2 (9.5)	0	1 (50.0)	0	1 (9.1)	0	0
Candida tropicalis	2 (9.5)	0	0	1 (25.0)	1 (9.1)	0	0
Cryptococcus gattii	0	0	0	0	0	0	0
Cryptococcus neoformans	0	0	0	0	0	0	0
Fusarium	0	0	0	0	0	0	0
Rhodotorula	1 (4.8)	0	0	1 (25.0)	0	0	0

* 3 samples only had off-panel organisms identified by standard-of-care methods (1 sample: *C. metapsilosis* and *Trichosporon asahii*; 1 sample: *Saccharomyces cerevisiae*; 1 sample: *Cryptococcus laurentii*); therefore, the ePlex BCID-FP Panel results were ‘Not Detected’ for all targets.

Supplementary Table 4. Demographics of subjects with positive blood culture for fungal organisms

	Prospectively-Collected (N=21)	Retrospectively-Collected (N=120)	All Clinical Samples (N=141)
Sex			
Male	14 (66.7)	68 (56.7)	82 (58.2)
Female	7 (33.3)	52 (43.3)	59 (41.8)
Age (years)			
Mean	48.1	53.5	52.7
Median	55.0	58.0	57.0
SD	22.4	22.5	22.5
Minimum	0.8	0.0	0.0
Maximum	90.0	89.0	90.0
<1 yr	1 (4.8)	2 (1.7)	3 (2.1)
1-17 yrs	2 (9.5)	8 (6.7)	10 (7.1)
18-44 yrs	4 (19.0)	27 (22.5)	31 (22.0)
45-64 yrs	11 (52.4)	39 (32.5)	50 (35.5)
65-84 yrs	2 (9.5)	39 (32.5)	41 (29.1)
85+ yrs	1 (4.8)	5 (4.2)	6 (4.3)

Supplementary Table 5. Blood Culture Bottle Type

	Contrived (N=725 [a]) n (%)	Prospective Fresh (N=11) n (%)	Prospective Frozen (N=10) n (%)	Retrospective (N=120) n (%)
BacT/Alert - SA (Standard Aerobic)	0	0	2 (20.0)	5 (4.2)
BacT/Alert - SN (Standard Anaerobic)	0	0	0	4 (3.3)
BacTec - Lytic/10 Anaerobic/F	0	0	1 (10.0)	19 (15.8)
BacTec - PLUS Aerobic/F	39 (5.4)	9 (81.8)	5 (50.0)	39 (32.5)
BacTec - PLUS Anaerobic/F	0	0	1 (10.0)	0
BacTec - Peds Plus /F	46 (6.3)	1 (9.1)	0	8 (6.7)
BacTec - Standard/10 Aerobic/F	0	0	0	27 (22.5)
BacTec - Standard Anaerobic/F	0	0	1 (10.0)	1 (0.8)
BacTec - Myco/F Lytic	640 (88.3)	0	0	0
VersaTREK - Redox 1 EZ Draw	0	1 (9.1)	0	17 (14.2)

[a] Only including the evaluable samples. One contrived sample was excluded due to a final, invalid result.
