# Supplementary material for: Multicenter Evaluation of a PCR-Based Digital Microfluidics and Electrochemical Detection System for the Rapid Identification of 15 Fungal Pathogens Directly from Positive Blood Cultures
Source: J Clin Microbiol. 2020 Apr 23;58(5):e02096-19. doi: 10.1128/JCM.02096-19 (PMC7180249; doi:10.1128/JCM.02096-19)
Supplement: Supplemental file 2 [file JCM.02096-19-s0002.pdf]

Supplementary Figure 1. ePlex BCID-FP Panel Report (IVD Report Shown)

GenMark Diagnostics | 5964 La Place Ct, Carlsbad, CA 92008

BCID-FP Detection Report

**Accession ID:**	555666777888	**Date/Time Completed:**	08/08/2019 11:29 AM
**Patient ID:**		**Bay Location:**	A1
**Protocol:**	BCID-FP 6.0.1.5	**Cartridge ID:**	12345614062
**Software Version:**	2.5.1.0	**Cartridge Lot Number:**	5162048
**Operator:**	Internal	**Cartridge Expiration Date:**	12/31/2020
**Instrument Serial Number:**	000000		

SUMMARY

No targets detected.

RESULTS

Target	Result
Candida albicans	Not Detected
Candida auris	Not Detected
Candida dubliniensis	Not Detected
Candida famata	Not Detected
Candida glabrata	Not Detected
Candida guilliermondii	Not Detected
Candida kefyr	Not Detected
Candida krusei	Not Detected
Candida lusitaniae	Not Detected
Candida parapsilosis	Not Detected
Candida tropicalis	Not Detected
Cryptococcus gattii	Not Detected
Cryptococcus neoformans	Not Detected
Fusarium	Not Detected
Rhodotorula	Not Detected
Internal Control	PASS

COMMENTS

FLAGS

Operator Signature Date

Approver Signature Date
